# Rheological study of Hall current and slip boundary conditions on fluid–nanoparticle phases in a convergent channel

**DOI:** 10.1039/d3na00616f

**Published:** 2023-10-24

**Authors:** Mubbashar Nazeer, M. Ijaz Khan, Sherzod Abdullaev, Fuad A. Awwad, Emad A. A. Ismail

**Affiliations:** a Department of Mathematics, Institute of Arts and Sciences, Government College University Faisalabad Chiniot Campus 35400 Pakistan mubbasharnazeer@gcuf.edu.pk; b Department of Mechanics and Engineering Science, Peking University Beijing China 2106391391@pku.edu.cn; c Department of Mechanical Engineering, Lebanese American University Beirut Lebanon; d Faculty of Chemical Engineering, New Uzbekistan University Tashkent Uzbekistan; e Department of Science and Innovation, Tashkent State Pedagogical University Named After Nizami Bunyodkor Street 27 Tashkent Uzbekistan sherzodbek.abdullaev.1001@gmail.com; f Department of Quantitative Analysis, College of Business Administration, King Saud University P.O. Box 71115 Riyadh 11587 Saudi Arabia fawwad@ksu.edu.sa emadali@ksu.edu.sa

## Abstract

*Purpose*: the purpose of this theoretical study was to analyze the heat transfer in the fluid–particle suspension model under the effects of a porous medium, magnetic field, Hall effects, and slip boundary conditions in a convergent channel with the addition of electrokinetic phenomena. The Darcy–Brinkman (non-Darcy porous medium) model was used to assess the effects of the porous medium. *Methodology*: the rheological equations of both models were transformed into a dimensionless form to obtain the exact solutions of the fluid and particle phase velocities, pressure gradient, volumetric flow rate, stream function, temperature distribution, and heat-transfer rate. To obtain an exact solution to the models, the physical aspects of the parameters are discussed, analyzed, and reported through graphs, contour plots, and in tabular form. *Findings*: mixing in hafnium particles in a viscous fluid provide 1.2% more cooling compared to with a regular fluid. A reduction of the streamlines was observed with the contribution of the slip condition. The utilization of the Darcy parameters upgraded both the fluid flow and temperature profiles, while the heat-transfer rate decreased by up to 3.3% and 1.7% with the addition of a magnetic field and porous medium, respectively. *Originality*: the current study is an original work of the authors and has not been submitted nor published elsewhere.

## Abstract 2

The purpose of this theoretical study was to analyze the heat transfer in the fluid–particle suspension model under the effects of a porous medium, magnetic field, Hall effects, and slip boundary conditions in a convergent channel with the addition of electrokinetic phenomena.

## Graphical abstract

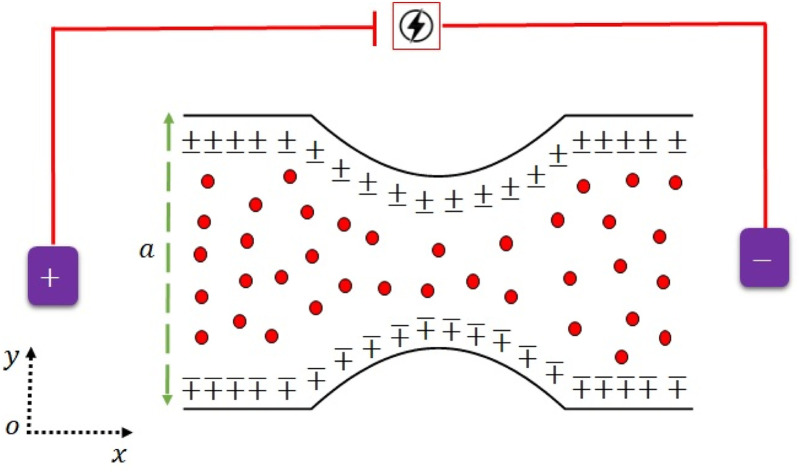

## 1. Introduction

Colloidal suspensions of nanoparticles generated by carbides, metal oxides, *etc.* appearing in a regular fluid (such as water, glycol, oil, and ethylene) can form a nanofluid. Much research is devoted to the search for different types of nanoparticles that have good mechanical properties (*i.e.*, thermal conductivity, a certain size of the particles, low particle momentum, and high mobility) to promote the heat-transfer analysis of the systems. Due to the distinguished properties of nanoparticles to increase heat transfer, nanofluids are used in various scientific processes (such as electronics, nuclear reactors, and biomedicine). The nanoparticles are also effectively used in many chemical processes and modern biotechnology (such as, artificial heart surgery, cancer therapy, drug delivery, sensor technology, disease diagnosis, and brain tumor therapy, due to their extensive thermophysical properties. Various researchers have performed work on different types of nanoparticles and base fluids in many diverse shapes of geometries to highlight the applications of nanofluids. For instance, Ellahi *et al.*^1^ reported the applications of nanoparticles in a cooling process. They considered the spherical shape of aluminum nanoparticles in kerosene oil (as a base fluid) with a maximum volume fraction of 4% to promote their applications in the cooling process. Bhatti *et al.*^2^ used a suspension of cobalt oxide and graphene nanoparticles in a carrier fluid to examine the applications of nanofluids in solar energy. They employed a successive linearization method to obtain numerical solutions to the problem. Their findings revealed that both nanoparticles could upgrade the heat-transfer rate, while skin friction displayed the opposite behavior. Hussain *et al.*^3^ suspended gold nanoparticles in a couple of stress fluids to discover their applications in gland and tumor remedies. They used a semi-analytical technique to obtain computational results for their model. Their results revealed that gold nanoparticles are the best option to kill the affected cells of tumors or glands due to their larger atomic number. Xu *et al.*^4^ showed the brilliant advantages of ultrasmall-sized nanoparticles in biomedical engineering (tumor therapy) and showed that nanoparticles sized less than 7 nm were more effective in kidney and tumor treatment compared to larger-sized nanoparticles (100–200 nm). Aljohani *et al.*^5^ chose a fractional derivative approach to show the applications of different types of nanoparticles in solar collectors to store solar energy. Some important results of nanofluids in different configurations were also reported by Dharmaiah *et al.*^6–8^ and Vedavathi *et al.*^9^

Fluid and heat transfer in a porous medium are attracting increasing interest from researchers due to their wider applications in geothermal systems, food industries, the insulation of buildings, the design of nuclear reactors, the manufacturing of thermal isolators, oil production, solar power reactors, hot rolling, drying technologies, the control of pollutant spread in groundwater, and in compact heat exchangers,^10–13^*etc.* Various models have been proposed by different authors to simulate the porous medium effects, such as Darcian and non-Darcian models, and non-equilibrium models. In 1856, Darcy proposed the porous medium model for assessing fluid flow through a porous medium experimentally by considering the linear relationship between the drop in pressure and the flow rate. The extensive body of literature suggests the importance of the porous medium in heat and flow analysis. For instance, Al Hajri *et al.*^14^ analyzed the importance of the porous medium in the heat-transfer analysis of a Maxwell fluid through a square conduit. The applications of a porous medium with heat transfer along a stretched cylinder were provided by Reddy *et al.*^15^ Asghar *et al.*^16^ used the Sisko fluid model to discuss the heat transfer with the porous medium in a curved channel by using the implicit finite difference method. Ramesh^17,18^ chose a non-uniform tilled channel model to discuss peristaltic flow under the influence of a porous medium and presented an exact solution to the problem.

Various engineering applications of heat transfer with porous media have been observed where cooling or heating is a very important factor, such as combustion systems, the cooling of turbine blades, the cooling of electronic devices, chemical reactors, storage in thermal-transport systems, and composite fabrication. The mixtures of low or high thermal fluids that appear in such applications can affect the output of these devices. In this situation, this issue can be resolved, and the performance of these devices can be increased (*i.e.*, the heat transfer) by utilizing a porous medium with nanofluids. Zhao *et al.*^22^ conducted a study on the utilization of Mg gas infiltration to produce MgB2 pellets, incorporating micro-sized B and nanosized powders. Zhang *et al.*^23^ investigated the energy absorption of water jet penetration on the 2A12 aluminum alloy. Chen *et al.*^24^ focused on the significance of the first hidden-charm pentaquark in relation to its strangeness. Also, the applications of nanofluids with a porous medium in heat-transfer analysis were reported by Nabway *et al.*,^19^ Kasaeian *et al.*,^20^ Mahdi *et al.*,^21^ Hussain *et al.*,^25,26^ Ge-JiLe *et al.*^27^ Cai *et al.*,^28^ Chen,^29^ and Du *et al.*^30^ in recent works on the applications of nanofluids in different applied fields.

The influences of the slip boundary conditions on the heat-transfer analysis of a fluid–particle suspension of a magnetohydrodynamic (MHD) electro-osmotic flow of a rheological fluid through a convergent shape geometry with a porous medium and the Hall effect have not been considered yet. Yet, we were motivated investigate these because of the potential applications of the slip boundary conditions in electro-osmotic flow to aid the design of reliable microfluidic devices and to ensure the effective operation of such devices. Here, the problem of slip boundary conditions can have a significant role in the study of heat and flow analysis to characterize the behavior of micro- and nanofluids through a microchannel. Currently, “no-slip” conditions are commonly used in the problem of microfluidic flows, but a key limitation of the no-slip condition is that it may fail to solve micro-, nanoscale fluid problems (depending on the roughness interface and the fluid–solid interface interaction).^31^ Due to this reason, it is important to use the slip boundary conditions during the study of the flow of fluids in a microchannel. It was Navier who first presented the slip boundary condition, in which he reported a linear relationship between the wall shear rate and the velocity of the slip. After his development, different researchers presented different types of slip boundary conditions,^32^ but the Navier slip boundary conditions are most commonly used due to their easiness and reliability. This article reports the solution of a fluid–particle suspension of an MHD electro-osmotic flow with heat transfer analysis of a rheological fluid under the consideration of the slip boundary conditions, porous medium, and Hall effects through a convergent geometry. In prior research, the crystallographic orientation, precipitation, mechanical characteristics, and phase transformation of a Ni-rich NiTi alloy were investigated by Wang *et al.*^47^ The authors examined the effects of the deposition current for a dual-wire arc on the alloy's properties. In recent studies, Zhao^48^ and Zhang *et al.*^49^ also explored the applications of co-precipitated Ni/Mn shell-coated nano Cu-rich core structures and analyzed the microstructural properties of alkali-activated composite nanomaterials. Additionally, Lu *et al.*^50^ and Kong *et al.*^51^ emphasized the importance of thermo-electric thermal fluid flows and conducted microspectroscopy analysis under high pressure.

## 2. Mathematical analysis

**Fig. 1:**
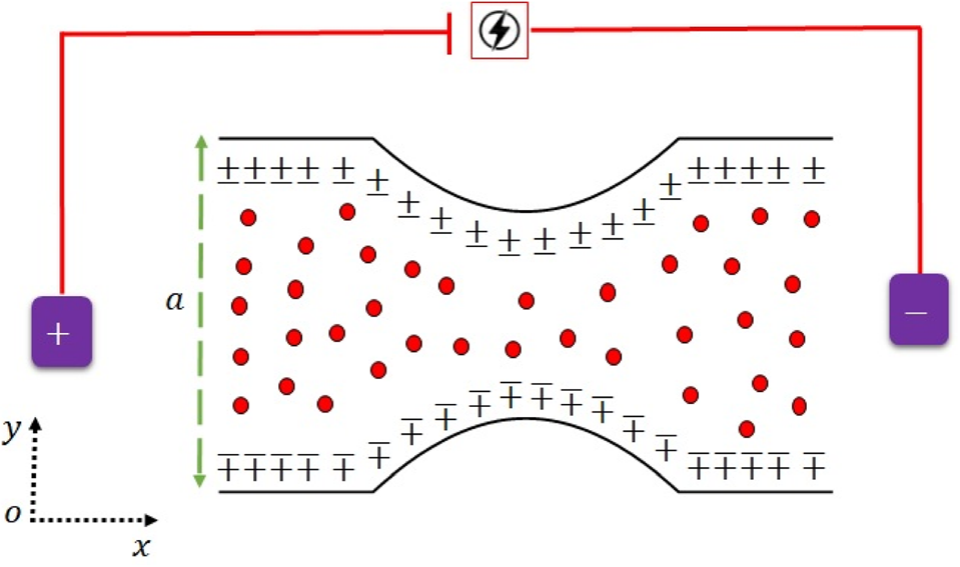
Physical sketch of the flow problem.

Consider the time-independent flow of a rheological fluid suspended by the addition of 40% hafnium solid spherical particles in as convergent channel as shown in Fig. 1. Here, **V**_vf_ = [*u*_vf_(*x*,*y*),*v*_vf_(*x*,*y*),0] and **V**_vp_ = [*u*_vp_(*x*,*y*),*v*_vp_(*x*,*y*),0] are the velocity vectors of the fluid and particulates.

The following assumptions are taken to simplify the given flow problem.

1. An external electric field *E*_*x*_ is applied along the *x*-axis direction.

2. The Debye–Hückel linearization approximation is applied.

3. The flow is considered in the Cartesian coordinate systems in which *x* and *y* are chosen in the axial and normal-to-flow directions, respectively.

4. The fluid is electrically conducted while the channel is non-electrically conducted.

5. A constant magnetic field *B*_0_ is applied normal to the flow direction.

6. The magnetic Reynolds number is very small, so the induced magnetic field is negligible.

7. The Hall current is considered while the ion slip effects and buoyancy force are neglected.

8. The flow and particle interact as a continuum.

9. The fluid and particle velocities are irrotational.

10. The Joule heating and thermal radiative heat flux effects are omitted.

11. The flow is symmetric along the center line of the channel *y* = 0 while *y* = *h*(*x*) and *y* = −*h*(*x*) are the upper and lower boundaries of the divergent channel, respectively.

12. The hafnium particles are considered to be in a spherical shape with equal size and uniformly distributed in the fluid.

13. The fluid and hafnium particles are coupled in terms of the drag force and heat transfer between them.

14. The top and lower walls of the channel maintain the temperatures *T*_0_ and *T*_1_, respectively.1
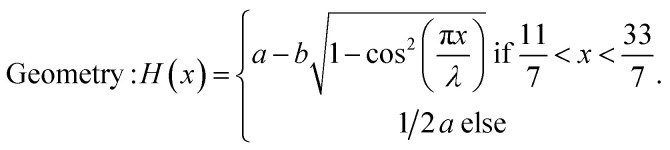


### 2.1 Physical model of the fluid phase

The rheological equations for the fluid phase in the vector form are defined as^33,34^2
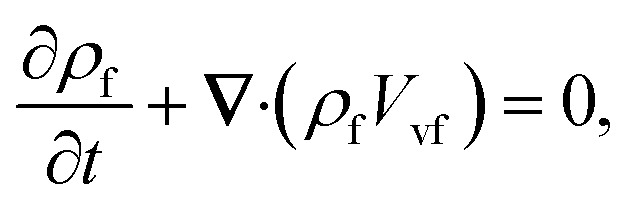
3
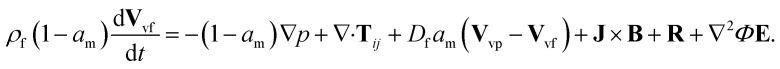


The Darcy resistance **R** = (*R*_*X*_,*R*_*Y*_,0), the electric current **J** = (*J*_*X*_,*J*_*Y*_,0), and the Cauchy stress tensor **T**_*ij*_ are expressed in the following form:4
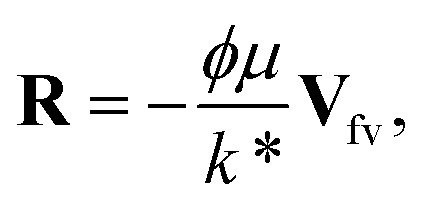
5
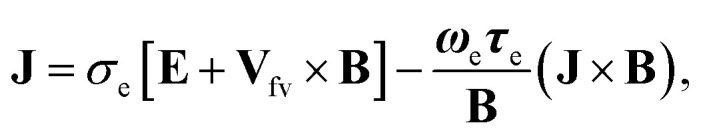

6 *T*_ij_*μ*_s_*A*, *A* = ∇*V* + (∇*V*)^T^.

Here *D*_f_(=6π*μr*) is called the Stoke drag coefficient, *r* is the radius of the hafnium particle, *φ*(0 < *φ* < 1) is known as the porosity of porous medium, k*(k* > 0) is the permeability of the porous medium, *σ*_e_ is the electrical conductivity, *ω*_e_ is the cyclotron frequency of electrons, *τ*_e_ is the electron collision time, **A** is known as the kinematical tensor, **E** is the electric field, and the total magnetic field is **B** = **B**_0_ + **b**, in which **B**_0_ is the applied magnetic field and **b** represents the induced magnetic field, which is assumed to be negligible due to taking a low magnetic Reynolds number. Here, we chose **E** = (0,0,0) = 0, due to the absence of applied polarization voltage. So the total uniform magnetic field with magnetic flux density is **B** = **B**_0_ = (0,0,*B*_0_). From the above assumption, eqn (5) can take the following form:^35–38^7
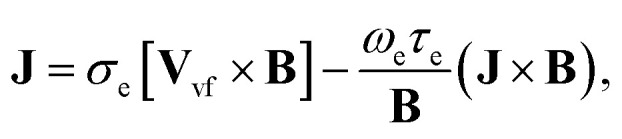


From the above eqn (7), we have

8 *J*_*x*_ = *σ*_e_*B*_0_*V*_vf_ − *m*_1_*J*_*y*_,

9 *J*_*y*_ = *σ*_e_*B*_0_*U*_vf_ − *m*_1_*J*_*x*_,…,

where *m*_1_ = *ω*_e_*τ*_e_ ≈ *O*(1) is called the Hall current parameter. Solving eqn (8) and (9) simultaneously, we get10
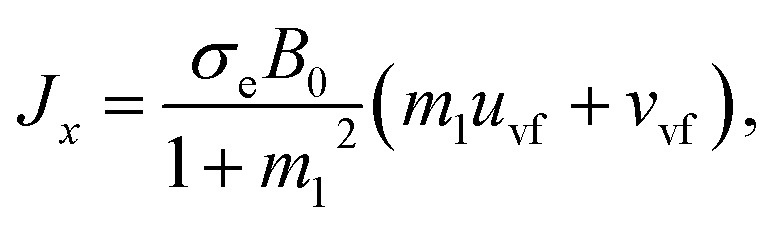
11
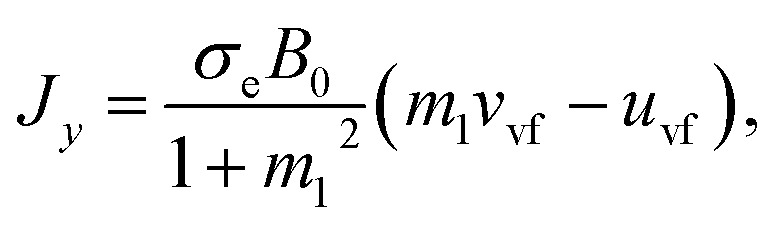


In eqn (10) and (11), *J*_*x*_ and *J*_*x*_ are the components of the current vector **J** in the *x*- and *y*- directions, respectively while *m* is the Hall current parameter.

### 2.2 Physical model of the particle phase

The rheological equations for the particle phase in vector form are defined as^39,40^12
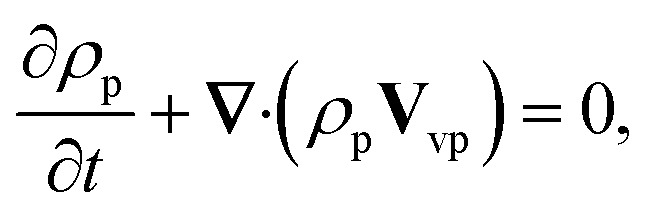
13
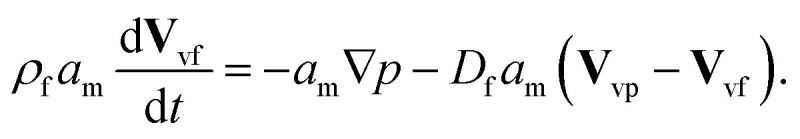


The vector form of the heat equation with viscous dissipation effects is defined as^41^14
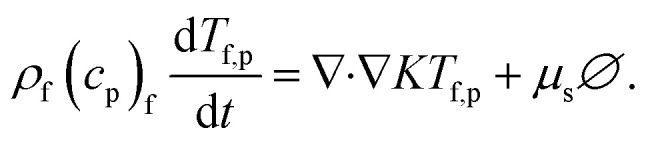


The components form of the fluid and particle phases flow equations are defined as15
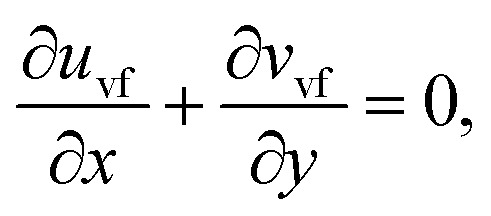
16
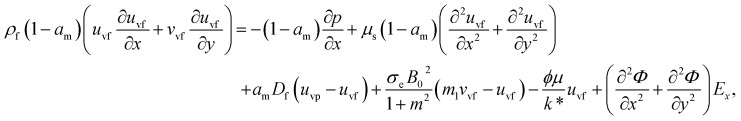
17
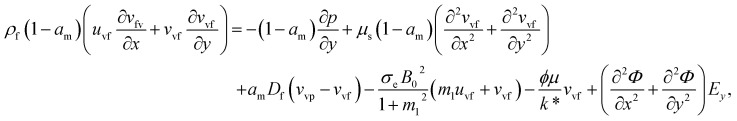
18
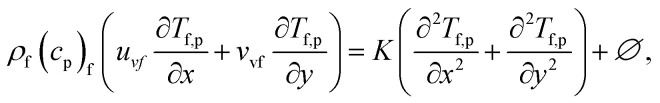
where the viscous dissipation term from the present flow problem is expressed as19
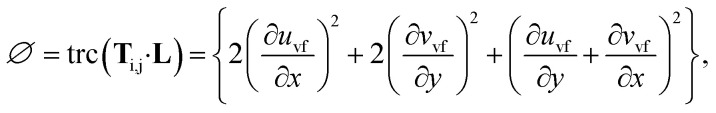
20
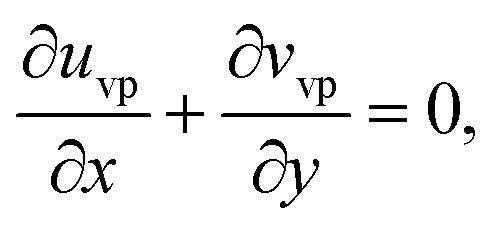
21
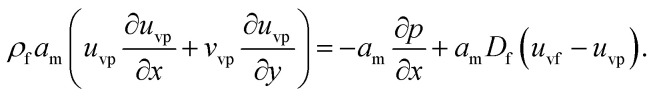


The boundary conditions are22
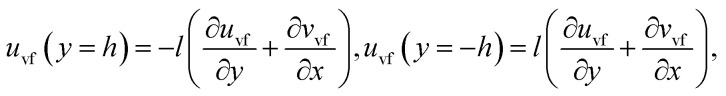
23
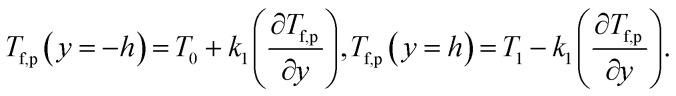


Introducing the dimensionless variable to convert the above-governing equations into dimensionless form24
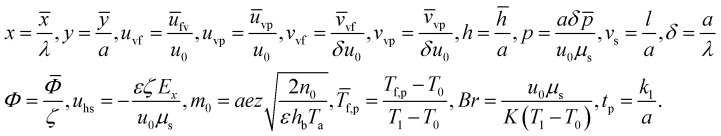


In view of the above equation, the dimensionless fluid–particle phase problem is expressed as (the bar has been removed)25
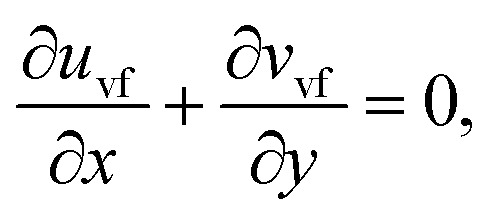
26
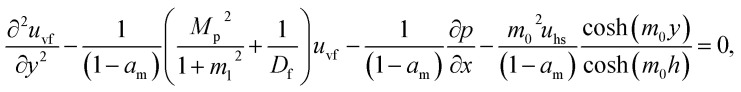
27
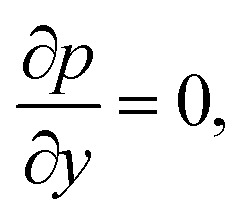
28
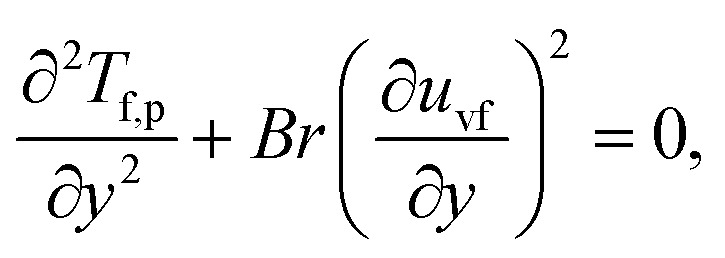
29
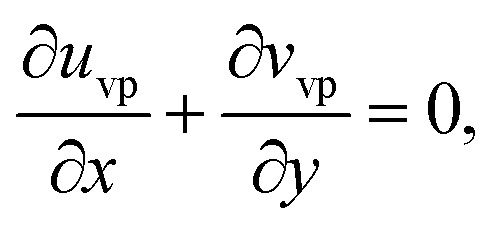
30
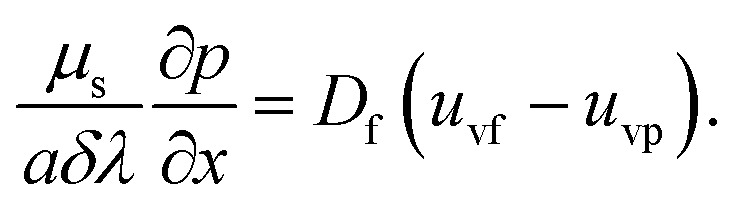


The boundary conditions are31
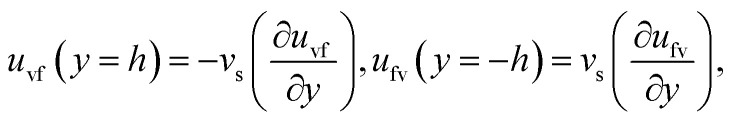
32
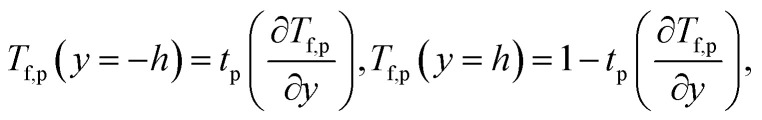
where33
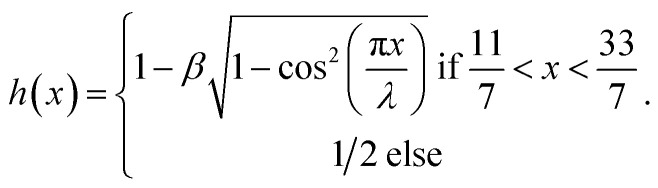


In eqn (24), 
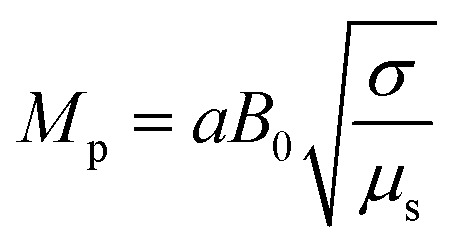
 is called the magnetic field parameter, 
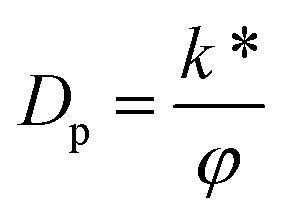
 is known as the porous medium parameter, *D*_f_ is the drag force, and Br is called the Brinkman number.

The exact solution, *i.e.*, the exact expressions for the fluid, particle, and temperature, is expressed as
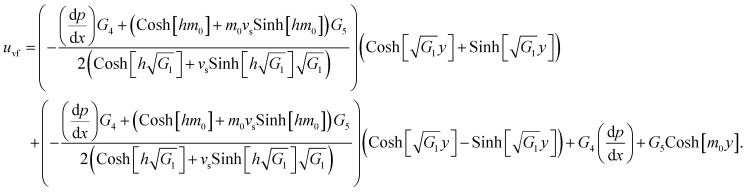

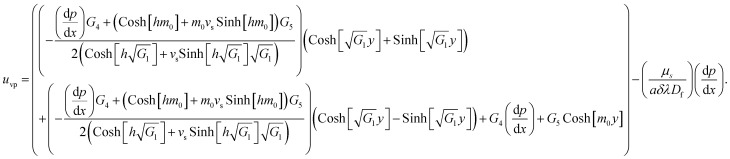


The expression of the total volumetric flow is obtained from the following mathematical expression as36
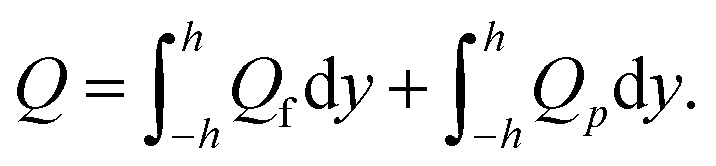
37
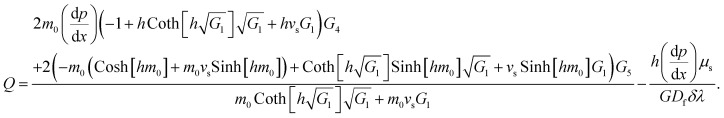


Solving the above equation for d*p*/d*x* gives38
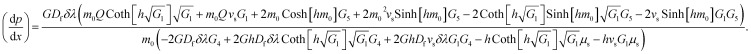


The expression of the stream function is calculated from the following mathematical relation 
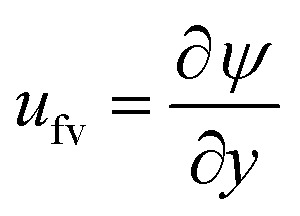
 and given as39
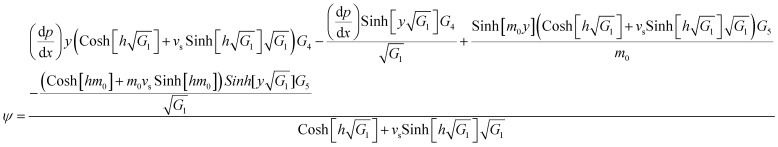


The expression of temperature distribution can be obtained from eqn (17) with boundary conditions (21)40
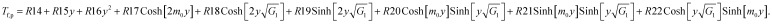


As an important physical quantity, the heat-transfer rate is calculated by using the relation 
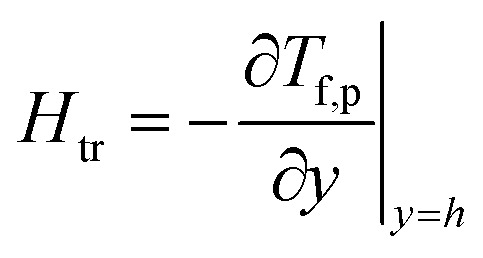
 and allows obtaining the following analytical expression:41
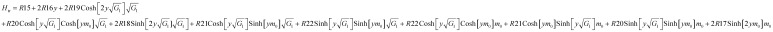


The constants *G*_1_,*G*_2_,*G*_3_,*G*_4_,*G*_5_, and *R*1,*R*2,…,*R*22 are defined in the appendix.

## 3. Particular cases

1. It is interesting to note that for a_m_ → 0, the problem is reduced to the single phase of an MHD electro-osmotic Newtonian fluid flow with heat transfer analysis in a convergent channel under the effects of slip boundary conditions, a porous medium, and Hall current without hafnium particles.

2. When *m*_1_ → 0, the problem is reduced to the multiphase electro-osmotic flow of a Newtonian fluid with heat-transfer analysis in a convergent channel under the effects of slip boundary conditions, a porous medium, and a magnetic field with a 40% suspension of Hafnium particles.

3. The finite value of *D*_p_ → ∞ corresponds to heat-transfer analysis of a biphase liquid flow of an MHD Newtonian fluid under the action of the Hall current and slip boundary conditions in a convergent channel with a 40% suspension of hafnium particles.

4. For *m*_0_ → 0, the problem is transformed into a simple multiphase pressure-driven flow of an MHD Newtonian fluid with heat-transfer analysis under the impact of the Hall current and slip boundary conditions in a convergent channel with a 40% suspension of hafnium particles.

5. When *Ha* → 0, the problem is reduced to the thermal transport of an MHD Newtonian fluid with a porous medium in a fluid–particle suspension through a convergent channel under the influence of the slip boundary conditions and Hall current.

6. For *v*_*s*_ = *t*_*p*_ = 0, the problem is reduced to thermal analysis of an MHD Newtonian fluid in a fluid–particle suspension in convergent channels without wall properties.

7. When *D*_p_ → ∞ and *m*_1_ = 0, the results of Hussain *et al.*^33^ and Ellahi *et al.*^34^ can be recovered, *i.e.*, the flow of a Newtonian nanofluid through a convergent channel without a heat-transfer mechanism.

## 4. Results and discussion

**Fig. 2:**
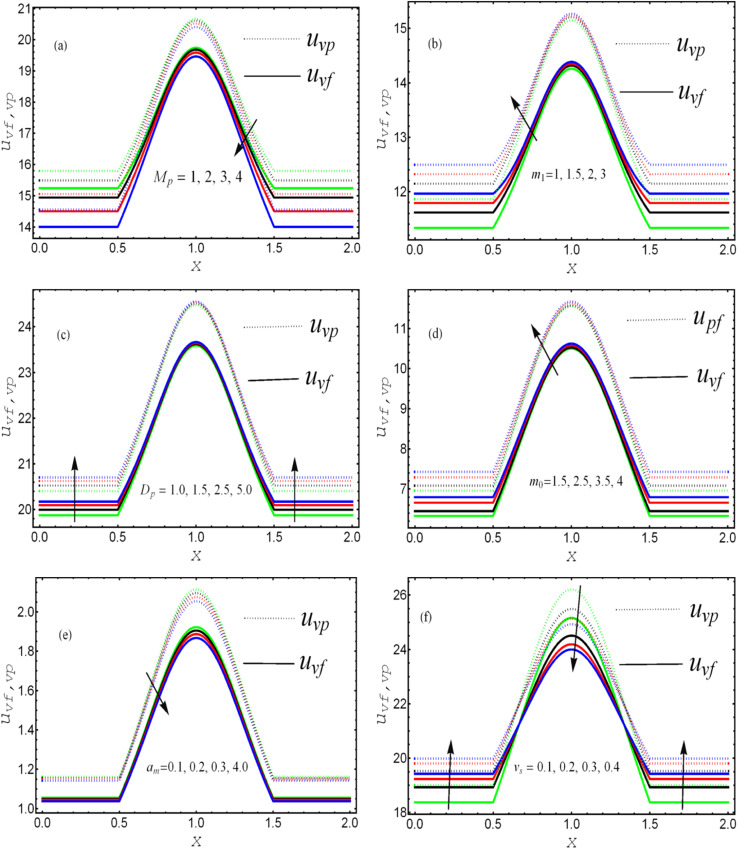
. Comparative analysis between the fluid and particle phase velocities against different values of (a) magnetic parameter, (b) Hall parameter, (c) Darcy parameter, (d) Debye length parameter, (e) coefficient of volume fraction, and (f) velocity slip parameter.

**Fig. 3:**
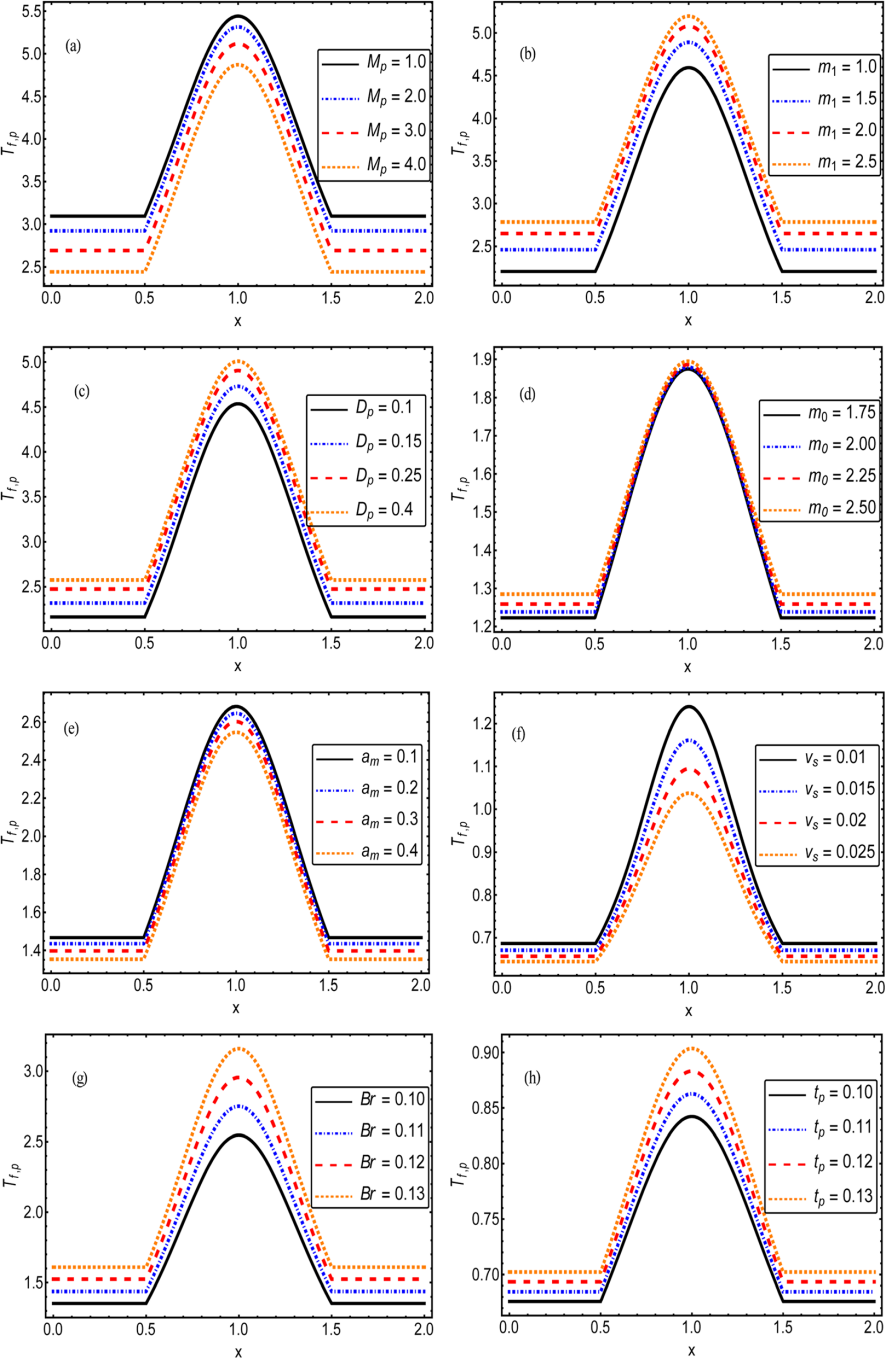
. Variation of the fluid and particle phase temperature against the different values of (a) magnetic parameter, (b) Hall parameter, (c) Darcy parameter, (d) Debye length parameter, (e) coefficient of volume fraction, (f) velocity slip parameter, (g) Brinkman number, and (h) thermal slip parameter.

**Fig. 4:**
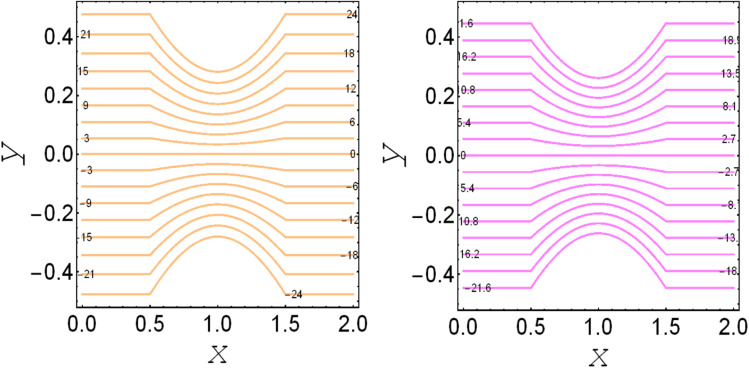
. Streamlines for *M*_p_ = 0.0 and *M*_p_ = 1.0.

**Fig. 5:**
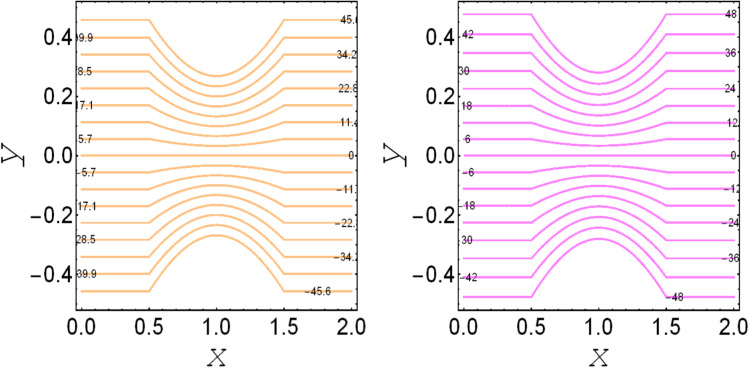
. Streamlines for *D*_p_ → ∞and *D*_p_ = 1.0.

**Fig. 6:**
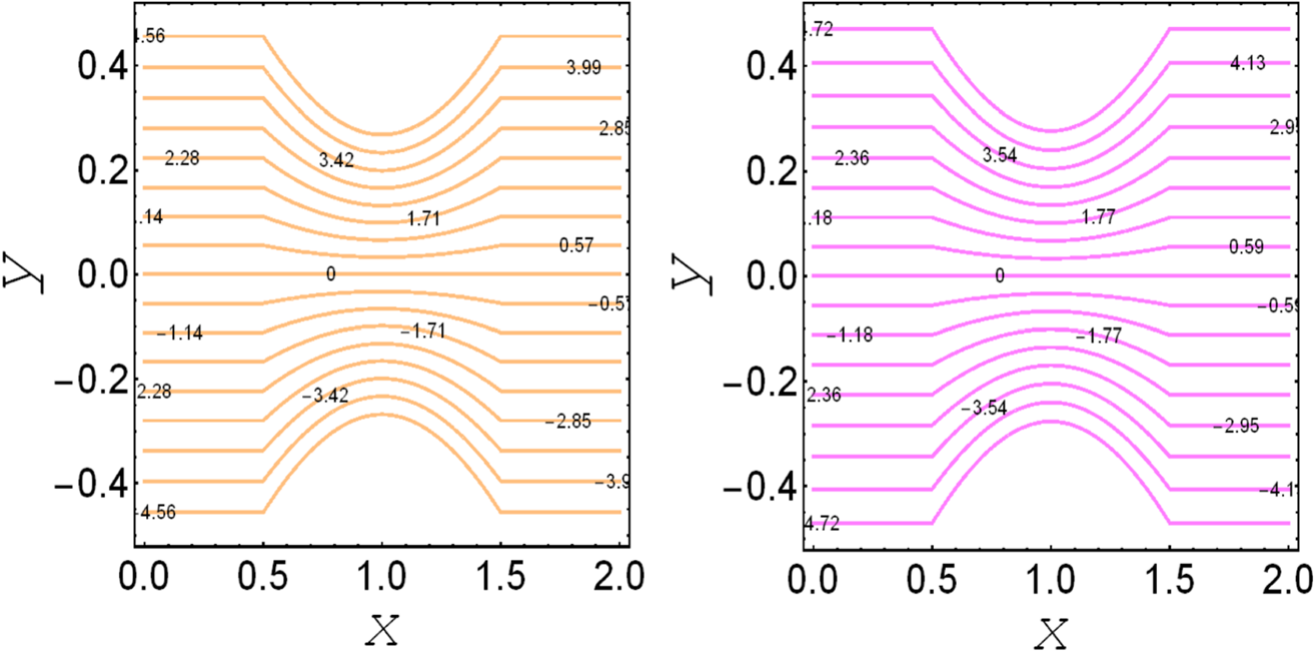
. Streamlines for *m*_1_ = 0.0 and *m*_1_ = 1.0.

**Fig. 7:**
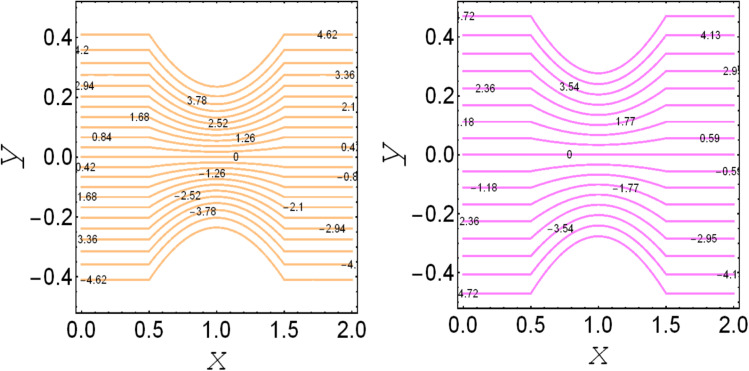
. Streamlines for *v*_s_ = 0.0 and *v*_s_ = 1.0.

**Fig. 8:**
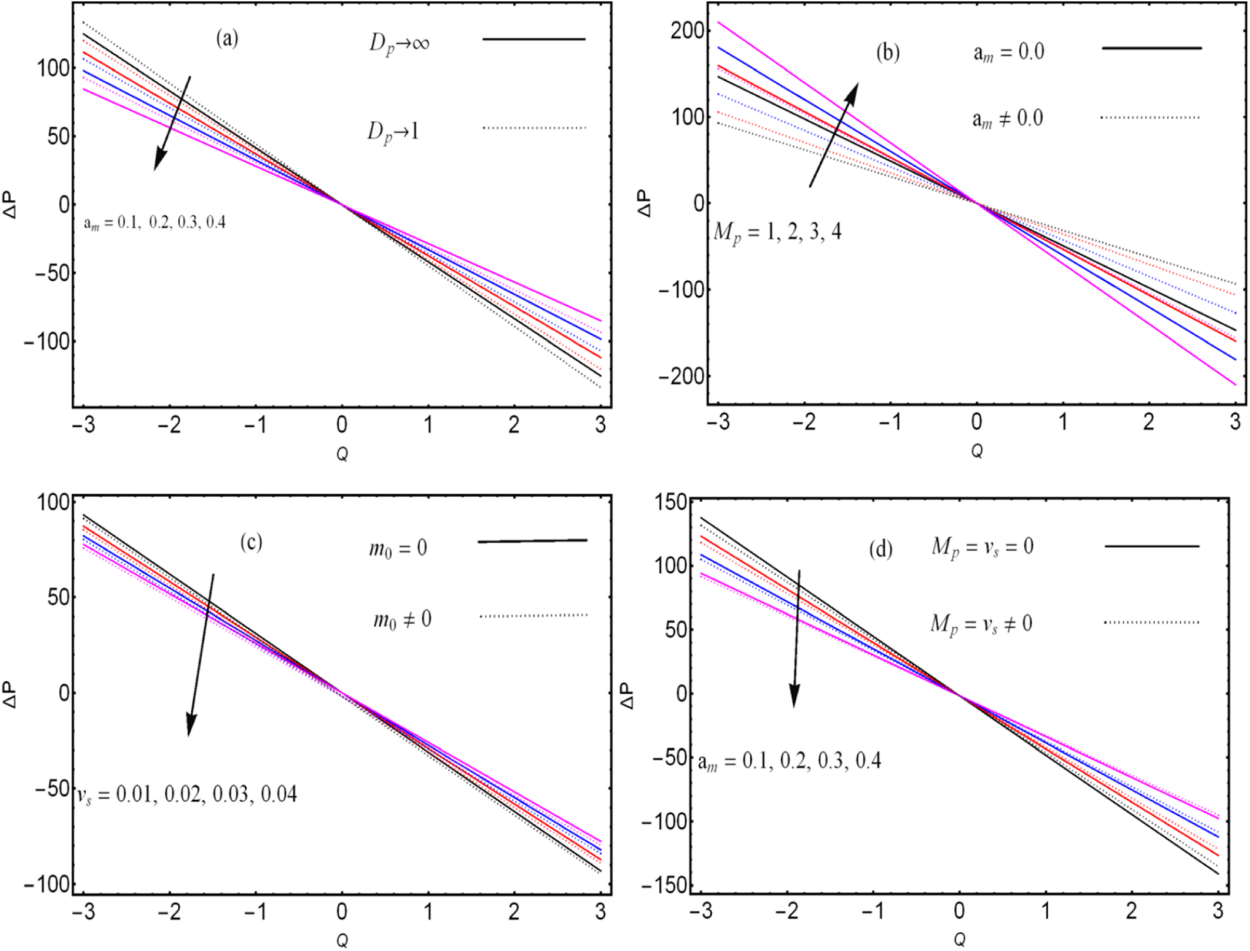
. Variation of the pressure rise (Δ*P*) against the different physical parameters.

In this section, we analyze the graphical behavior of the important physical quantities, namely the fluid and particle velocities, stream function, temperature distribution, and heat-transfer rate under the involved parameters of the study, namely the Debye length parameter 
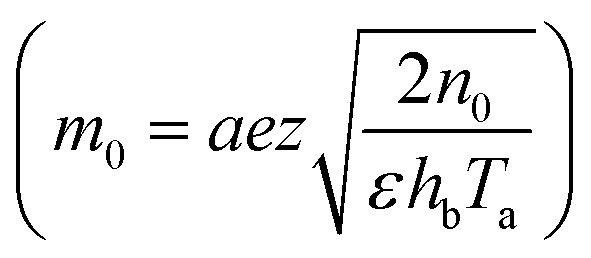
, magnetic parameter 
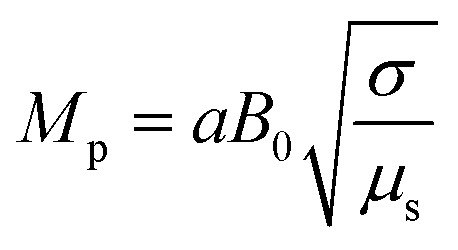
, Hall parameter (*m*_1_ = *ω*_*e*_*τ*_*e*_), velocity slip parameter 
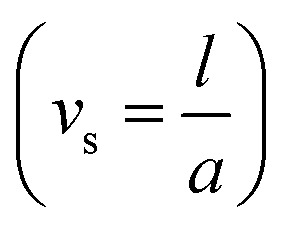
, Darcy parameter 
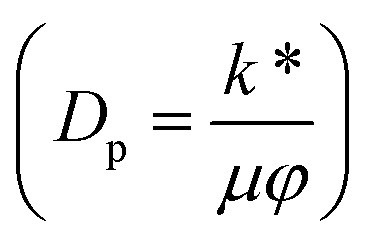
, Brinkman number 
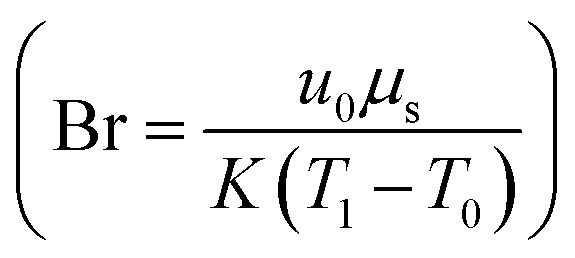
, particle suspension parameter (a_m_), and thermal slip parameter 
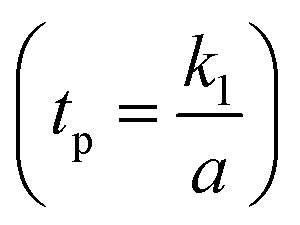
. The solution to the considered problem is obtained with the help of mathematical software and presented as closed-form expressions of the fluid and particle velocity, stream function, total volumetric flow rate, pressure gradient, temperature distribution, and local heat-transfer rate. The range of the involved parameters for computation results is listed in Table 1. The author present seven figures. Fig. 2 shows the variation of the fluid and particle velocities, while Fig. 3 reports the temperature distribution, Fig. 4–7 show the stream function behavior, and the graphs of the pressure rise are displayed in Fig. 8. The variation of heat-transfer rate is presented in Table 2.

**Table 1:** Range of the physical parameters used in the current computational analysis

Name	Notation	Range	Reference
Debye length parameter	*m*_0_	[0,10]	42
Hall parameter	*m*_1_	[0,3]	43
Thermal slip parameter	*t*_p_	[0,0.1]	44
Brinkman number	Br	[0,10]	27
Velocity slip parameter	*υ*_s_	[0,0.1]	44
Magnetic field parameter	*M*_p_	[0,5]	45
Darcy parameter	*D*_p_	[0,∞]	10–13
Electro-osmotic velocity	*U*_hs_	[0,10]	33 and 34
Coefficient of particle fraction	*α*_m_	[0,0.6]	46

**Table 2:** . Variation of the heat-transfer rate against the following parameters *U*_hs_ = 5, *Q* = 50

*α*_m_	*υ*_s_	*t*_p_	*m*_0_	*m*_1_	Br	*D*_p_	*M*_p_	*H*_tr_	%	Behavior
0.0	0.1	0.1	1.0	1.0	0.1	1.0	1.0	4.9197	1.2	Decrease
0.4	—	—	—	—	—	—	—	4.8614		
2.0	0.0	—	—	5.0	—	—	—	14.4155	8.7	Decrease
—	0.01	—	—	—	—	—	—	13.1587		
—	0.1	0.0	—	—	—	—	—	7.0139	2.4	Increase
—	—	0.1	—	—	—	—	—	7.1805		
—	—	—	0.0	—	—	—	—	7.0904	1.3	Increase
—	—	—	1.0	—	—	—	—	7.1805		
—	—	—	—	0.0	—	—	—	4.8884	2.6	Increase
—	—	—	—	2.0	—	—	—	5.0152		
—	—	—	—	1.0	0.1	—	—	16.7042	53.8	Increase
—	—	—	—	—	0.15	—	—	25.6926		
—	—	—	—	—	0.1	∞	—	4.9851	1.7	Decrease
—	—	—	—	—	—	1.0	—	4.8979		
—	—	—	—	—	—	1.0	0.0	4.9408	3.3	Decrease
—	—	—	—	—	—	—	2.0	4.7768		

The effects of the magnetic field parameter (*M*_p_) on both the fluid and particle velocities are displayed in Fig. 2(a). The dashed and solid lines are used in the graphs to differentiate the particle and fluid velocity distribution, respectively. This figure shows that increasing the magnetic field parameter led to decreases in both velocities in the divergent channel, and this behavior was due to resistive force *i.e.*, Lorentz force (**J** × **B**). Basically, the greater the values of the magnetic field parameter, the larger the magnetic field produced relative to the viscosity of the fluid, which enhances the Lorentz force acting on the fluid particles and reduces the motion of the fluid particles. The influence of the Hall parameter on the velocity distribution is highlighted in Fig. 2(b). Here we observed that the Hall parameter and magnetic field had a direct and inverse relation on the velocity profiles, respectively, *i.e.*, the magnetic field parameter disturbed the fluid flow, which caused a reduction in the fluid velocity, while the Hall parameter supported the motion of the fluid particle, and the velocities distribution increased against it. Since *m*_1_ = *ω*_e_*τ*_e_, *i.e.*, the cyclotron frequency of electrons and the electron collision time have a direct relationship with the Hall parameter, which means that when the Hall parameter is increased, then the cyclothron frequency of the electrons in the fluid particles increases, which causes an increase in both velocities' distribution. This can also be expressed by another mathematical relation 
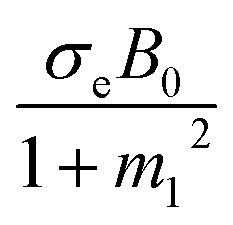
, *i.e.*, the greater values of the Hall parameter diminish the electrical conductivity, which causes a reduction in the damping force, and as a result the velocity of both phases increases against the Hall parameter. Fig. 2(c) shows the increasing behavior of the velocity distribution against the Darcy parameter (*D*_p_). Physically, this means that the Darcy parameter reduces the drag force in the fluid particles, which causes an enhancement in the velocity profile *via* the Darcy parameter. We can also express that, for increasing values of the Darcy parameter, a more permeable porous medium will exist, which will provide less resistance to the fluid particles as a result of the velocities profiles enhancement. The effects of the Debye length parameter (*m*_0_) on the velocities are examined in Fig. 2(d), in which the increasing trend of the velocities distribution is captured against this parameter. Since the Debye length parameter 
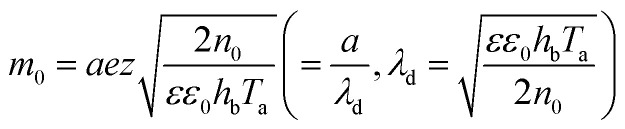
, *i.e.*, the Debye length parameter is defined as “the height of the channel (*a*) over the Debye thickness (*λ*_d_)”, when the height of the channel increases, then the velocity distribution will increase, and as a result an electrical double layer arises. The effects of the coefficient of the volume fraction on velocity profiles are shown in Fig. 2(e), in which the velocity profiles decrease when increasing the coefficient of the volume fraction. From the computational results, it was observed that the velocity of the clean/simple fluid was greater than the velocity of the multiphase fluid (*i.e.*, the fluid with the suspension of hafnium particles). The physical reason for this is that when the solid tiny particles of hafnium are mixed in simply, then these particles generate internal friction in the resultant fluid (multiphase fluid), which retards the fluid flow. Fig. 2(f) was constructed to analyze the impact of the velocity slip parameter on the fluid–particle phase velocities. From this figure, it can be noted that the velocity of both phases showed a mixed behavior *via* the velocity slip parameter, *i.e.*, the velocity profiles increased and decreased in the interval *x* ∈ [0,0.65]*U*[1.35,2.0] and *x* ∈ [0.65,1.35], respectively.

The impact of the magnetic field parameter 
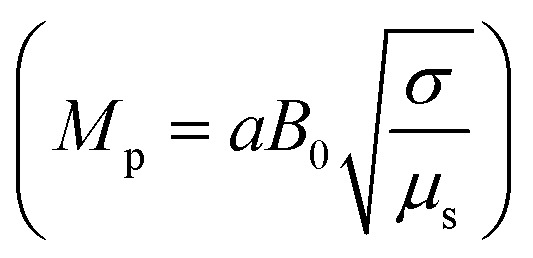
 on the temperature distribution is illustrated in Fig. 3(a), which shows the inverse relationship between the temperature profile and the magnetic field parameter. It could be observed that the hafnium particles lost their temperature when the magnetic force was applied, and we can also say that the reduction in temperature distribution happened due to departing effects. Fig. 3(b) depicts the influence of the Hall parameter (*m*_1_ = *ω*_e_*τ*_e_) on the temperature profile, and we could observe that this parameter enhanced the temperature distribution, and the maximum temperature was noted to be in the center of the channel. The variations of temperature against the Darcy parameter 
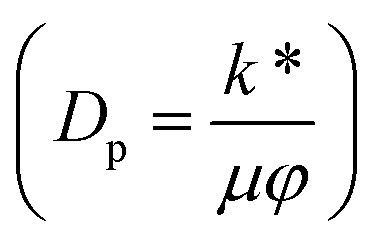
 and Debye length parameter 
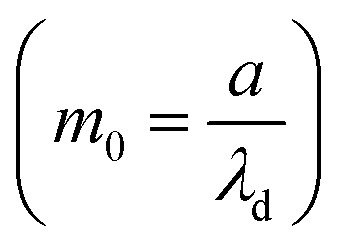
 are shown in Fig. 3(c) and (d), respectively. Both figures indicate the direction variation of the temperature profiles with these parameters. Fig. 3(e) and (f) exhibit the impact of the coefficient of the volume fraction (a_m_) and velocity slip parameter 
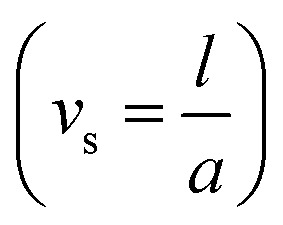
 on the temperature of the fluids and particles, respectively. Both figures show the decreasing behavior of the temperature distribution *via* the coefficient of the volume fraction and velocity slip parameter. The physical reason is that when hafnium particles are suspended in the fluid, they absorb the heat, and as a result, the temperature decreases. Thus, we can say that the viscous fluid with the suspension of hafnium particles plays a significant role in the cooling process compared to in a regular viscous fluid. Fig. 3(g) and (h) characterize the temperature behavior against the Brinkman number 
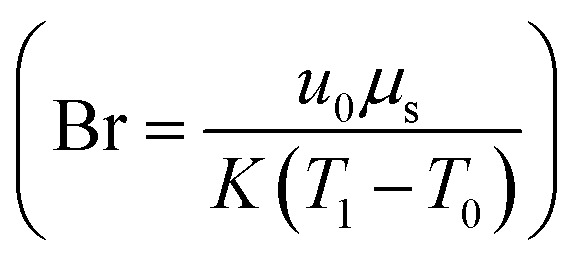
 and thermal slip parameter 
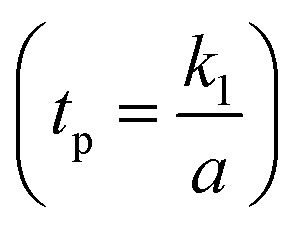
. These figures show increasing trends *via* considering the Brinkman number and thermal slip parameter. The physical reason for the increasing temperature against the Brinkman number is that when the Brinkman number increases, the viscous dissipation produces the condition of the heat-transfer mode, which enhances the temperature profile. The impacts of the magnetic parameter, Darcy parameter, Hall parameter, and velocity slip parameter on the stream function are captured in Fig. 4–7, respectively. From these figures, a significant change can be observed in the stream function graphs. The magnitude of the stream is reduced with the contribution of the magnetic field, which happened due to the Lorentz force produced against the applied magnetic field (see Fig. 4). On the other hand, the Darcy parameter controls the magnitude of the stream function, *i.e.*, the stream function obtains a greater magnitude with consideration of a porous medium as compared to a clear medium (see Fig. 5). A similar trend for the stream function was recorded against the Hall current parameter and velocity slip parameter (see Fig. 6 and 7). The variation of the heat-transfer rate under the effects of the parameters of the study is shown in Table 2. From this table, it can be noted that there was a 1.2% reduction in the heat-transfer rate in the case of the suspension with hafnium particles, suggesting that the hafnium particles absorb heat when they are suspended in viscous fluid. Thus, the hafnium particles provided 1.2% greater cooling when they were mixed in the viscous fluid compared to a regular fluid. The percentage reduction in heat transfer against the velocity parameter was 8.7%, which means that the velocity slip parameter controlled the heat transfer of the system *i.e.*, the slip parameter provided 8.7% more cooling compared to the case without slip conditions. The heat-transfer rate also decreased by up to 3.3% and 1.7% with the addition of a magnetic field and porous medium, respectively. These results suggest that these parameters also favor the cooling process of the system. The thermal slip parameter, Debye length parameter, Hall parameter, and Brinkman number thus support the heat-transfer rate, *i.e.*, these parameters increase the heat transfer rate when increasing these parameters, and the percentages heat transfers were 2.4%, 1.3%, 2.6%, and 53.8%, respectively, see Table 2. Thus, the thermal slip parameter, Debye length parameter, Hall parameter, and Brinkman number favor the heating system. Fig. 8 illustrates an analysis of the characteristics of the pumping phenomena. It could be observed that the pressure rise decreased in the region 0 < *Q* & 0 < Δ*P* (*i.e.*, the retrograde pumping region) and increased in the region *Q* > 0 & Δ*P* > 0 (*i.e.*, the co-pumping region) when increasing the coefficient of the volume fraction and the Darcy parameter (see Fig. 8(a)). The pressure rise also increased *via* the magnetic field parameter in the retrograde pumping region (*Q* > 0 & Δ*P* > 0) and decreased *via* the magnetic field parameter in the co-pumping region (0 < *Q* & 0 < Δ*P*), see Fig. 8(b). Fig. 8(c) examines the behavior of the pressure rise against the velocity slip parameter for *m*_0_ = 0 and *m*_0_ ≠ 0 and it could be noted that the pressure rise showed a similar trend against the velocity slip parameter and Debye length parameter, as can be seen in Fig. 8(a). Considering the characteristics of the pressure rising *versus* the coefficient of the volume fraction for the two different cases, *i.e.*, when the magnetic field and slip conditions are ignored, and when the magnetic field and slip conditions are included, we see that the pressure rise decreased in the region 0 < *Q* & 0 < Δ*P* (*i.e.*, the retrograde pumping region) and increased in the region *Q* > 0 & Δ*P* > 0 (*i.e.* the co-pumping region) when increasing the coefficient of the volume fraction (see Fig. 8(d)).

## 5. Solution validation

**Fig. 9:**
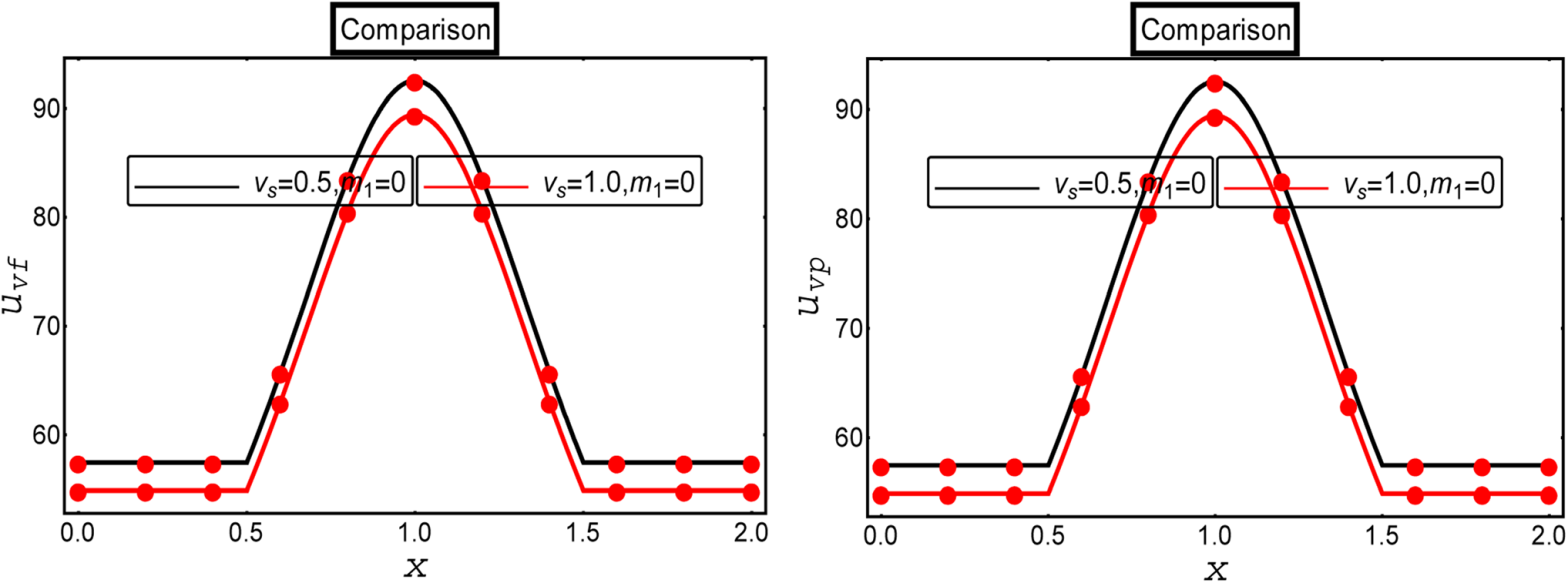
. Comparative analysis with the existing literature.

A comparative analysis was performed with the existing results available in the literature to verify the current calculated results. For this, we compared our results with the computational results of multiphase flow for a Newtonian fluid with a suspension of hafnium nanoparticles through a convergent channel as presented by Ellahi *et al.*^34^ Ellahi *et al.*^34^ developed the mathematical model of the two-phase flow of a Newtonian fluid through the diverse shape of the channel under the action of electro-osmotic and magnetic forces, and velocity slip conditions, and presented the exact solution through MATHEMATICA 12.0. The present results are in full agreement with the results of Ellahi *et al.*^34^ for the limiting case *m*_1_ → 0 and *D*_p_ → ∞. The variations of the fluid and particle phase velocities are illustrated in Fig. 9 under the effects of the velocity slip parameter. It could be observed that the impact of the velocity slip parameter in the current study (the red solid graph) was analogous to the results in the existing literature (black solid graph).

## 6. Concluding remarks

This study was conducted to analyze the heat-transfer rate of an MHD rheological fluid with electro-osmotic flow with a fluid and particle model through a convergent channel under the consideration of a porous medium, slip conditions, and Hall effects. The rheological equations were simplified and solved in the dimensionless form in MATHEMATICA software and exact solutions for the fluid–particle velocities, stream function, volumetric flow rate, pressure gradient, temperature distribution, and heat transfer rate were presented. Then to analyze the physical behavior of the important physical quantities, graphs and tables were constructed. The important findings are listed below:

• The Hall current parameters increased the velocity and temperature of the fluid–particle phases.

• The velocity slip parameter diminished the temperature distribution while the thermal slip parameter enhanced the temperature.

• Reduction of the streamlines was observed with the contribution of the slip condition.

• The Darcy parameter upgraded both the fluid and temperature profiles.

• The magnitude of the stream function increased against the Debye length parameter.

• The viscous fluid with a suspension of hafnium particles played a significant role in the cooling process compared to the regular viscous fluid.

• The heat-transfer rate decreased by up to 3.3% and 1.7% with the addition of a magnetic field and porous medium, respectively.

• The thermal slip parameter, Debye length parameter, Hall parameter, and Brinkman number support the heat-transfer rate, *i.e.*, these parameters increased the heat-transfer rate by increasing these parameters, and the percentage increases in terms of heat transfer were 2.4%, 1.3%, 2.6%, and 53.8%, respectively.

• The hafnium particles provided for 1.2% more cooling when they were mixed in the viscous fluid compared to the regular fluid.

• This study can be extended by utilizing the non-Newtonian fluid models with heat-transfer analysis and slip boundary conditions.

## Appendix


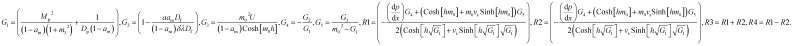
 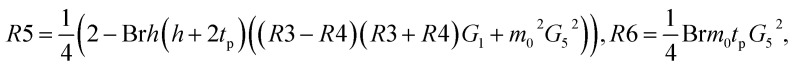 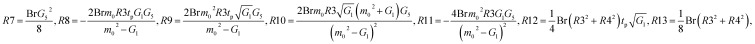 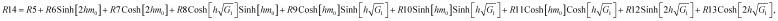 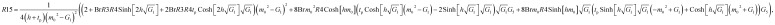 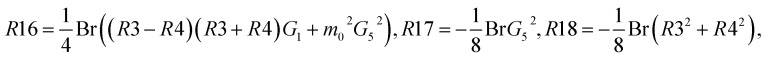 
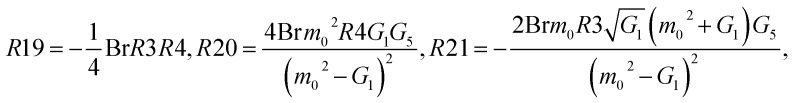
 
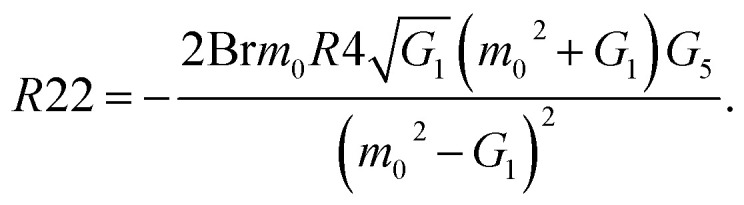


## Nomenclature

*H*_tr_
: The heat transfer rate
*b*
: The width of the channel (m)
*a*_m_
: The coefficient of particle fraction (kg m^3^)
*δ*
: The wave number
*D*_f_
: The drag force
*m*_0_
: The Debye length parameter (m)
*v*_s_
: The velocity slip parameter
Br: Brinkman number
*y*
: The coordinate axis (m)
*Q*
: Total volumetric flow rate
*Q*_p_
: The volumetric flow rate of particle velocity
*μ*_*s*_
: The viscosity kg m^−1^ s^−1^
*v*_vp_
: The component of particle velocity (m s−1)
*M*_p_
: The magnetic field parameter
*Φ*
: The electro-osmotic potential function (V)
*T*_0_
: The temperature of the lower wall (K)
*l*
: The length of the channel (m)
*ρ*_f_
: The density of the fluid (kg m^−3^)
*σ*_e_
: The electrical conductivity (s m^−1^)
∇: The dell operator
*D*_p_
: The Darcy parameter
*φ*
: The porosity of the porous medium
k*: The permeability of the porous medium (m^2^)
**V**_vp_
: The velocity vector of the particle phase (m s^−1^)

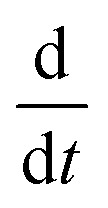

The material time derivative
*T*_a_
: The absolute temperature (K)
*E*_*x*_
: The component of the electric field in *x* direction (V m^−1^)
*k*_1_
: The thermal slip constant
ψ: The stream function (m^2^ s^−1^)
*ε*
: The permittivity of the fluid F m^−1^
*m*_1_
: The Hall parameter
*λ*
: The wavelength (m)
*u*_hs_
: The electro-osmotic velocity (m s^−1^)
*a*
: The wave amplitude (m)
t_p_: The thermal slip parameter
*x*
: The coordinate axis (m)
*h*
: The wall of the channel
*Q*_f_
: The volumetric flow rate of fluid velocity
*β*
: The amplitude ratio
*u*_vp_
: The component of fluid velocity (m s^−1^)
*T*_f,p_
: The temperature of fluid and particle phase (K)
*n*_0_
: The bulk ionic concentration
*K*
: The thermal conductivity (W mK^−1^)
*T*_1_
: The temperature of the upper wall (K)
*ω*_e_
: The cyclotron frequency of electrons
*B*_0_
: The strength of the magnetic field (Wb m^−2^)
*∅*
: The contribution of viscous dissipation term
*p*_*y*_
: The yield stress of the fluid
**T**_*ij*_
: The (*i*,*j*)^*th*^ components of the stress tensor
**V**_vf_
: The velocity vector of the fluid phase (m s^−1^)
*g*
: The gravitational force (m s^−2^)
*t*
: The time (s)
*τ*_e_
: The electron collision time
*h*_b_
: The Boltzmann constant
*E*_*y*_
: The component of the electric field in *y* direction (V m^−1^)
*p*
: The pressure (N m^−2^)

### Subscripts

f: The fluid phase
p: The particle phase
*v*_f_
: The fluid velocity
*v*_p_
: The particle velocity

## Conflicts of interest

The authors have no conflict of interest related to this study.

## Supplementary Material
